# Ectopic Pregnancy in Tigray, Ethiopia: A Cross-Sectional Survey of Prevalence, Management Outcomes, and Associated Factors

**DOI:** 10.1155/2021/4443117

**Published:** 2021-11-30

**Authors:** Elsa Tesfa Berhe, Kalayu Kiros, Merhawit Gebremeskel Hagos, Hailay Abrha Gesesew, Paul R. Ward, Teferi Gebru Gebremeskel

**Affiliations:** ^1^Department of Reproductive Health, College of Health Sciences, Aksum University, Aksum, Ethiopia; ^2^School of Medicine, College of Medicine and Health Science, Aksum University, Aksum, Ethiopia; ^3^Department of Midwifery, College of Health Sciences, Aksum University, Aksum, Ethiopia; ^4^Discipline of Public Health, Flinders University, Adelaide, Australia; ^5^Department of Epidemiology, College of Health Sciences, Mekele University, Mekele, Ethiopia

## Abstract

**Background:**

Ectopic pregnancy is a neglected and challenging gynecologic problem in developing countries including Ethiopia.

**Objective:**

The present study is aimed at assessing the prevalence of ectopic pregnancy, its management outcomes, and factors associated with management outcomes in Tigray, North Ethiopia.

**Methods:**

We employed a four-year retrospective cross-sectional study from September 2015 to August 2019. We extracted data about all pregnant mothers who were admitted and managed for EPs in Axum, Tigray. Ectopic pregnancy and its outcomes (favorable and unfavorable) were the dependent variables, and age, residence, ethnicity, religion, parity, history of abortion, history of EP, pelvic infections, history of surgical procedures, and use contraceptives were the independent variables. We employed descriptive statistics and bivariate and multivariate logistic regression analyses using SPSS. Ethical clearance was obtained from Axum University, Tigray, Ethiopia.

**Results:**

The overall prevalence of ectopic pregnancy was 0.52% of total deliveries, which equates to 1 : 193 deliveries. Surgery for ectopic pregnancy accounts for 7.6% of all gynecological surgeries. Most participants were in the age group 26–30 years and lived in rural areas. Among the different EP implantation sites, most cases (92.4%) occurred in the fallopian tube, followed by 5.1% in the ovary and 2.5% in abdominal EPs. Surgical management (laparotomy) was undertaken for all the 79 women diagnosed with EPs, including laparotomy (100%), salpingo-oophorectomy (17.7%), salpingectomy (73.9%), oophorectomy (3.4%), cornual resection (2.5%), and removal of concepts tissue 2.5. The record reports that intraoperative procedure was correctly managed for 47 (59.5%) women but the condition of EP procedure was ruptured for about two-thirds (63.3%) of the women. Thirty (38%) patients had developed some complications after surgery including anemia (hemoglobin < 10.5) (*n* = 12), fever (*n* = 10), wound infection (*n* = 2), and pneumonia (*n* = 2). Women who were from urban (AOR = 11.2, 95% CI: 2.65-47.2) and who had normal hemoglobin at presentation (AOR = 9.94, 95% CI: 2.03-48.7) were associated with favorable maternal outcomes.

**Conclusions:**

More than one-third of women with ectopic pregnancies had an unfavorable maternal outcome, which was higher among rural residents and anemic mothers. Women living in rural areas and anemia during pregnancy should seek special attention in the management of EPs. We also recommend improving the data management of hospitals in Ethiopia.

## 1. Introduction

Ectopic pregnancy (EP), an extrauterine pregnancy, is one in which the blastocyst implants anywhere other than the endometrial lining of the uterine cavity [1]. EP is a global problem, and the most common life-threatening emergency in gynecological practice and is a medical emergency that requires immediate treatment [2]. This complication is prevalent in low- and middle-income countries, where the majority of mothers present late after their onset of illness with hemodynamic instability and tubal rupture [3]. A five-year retrospective study (2009-2013) in the University of Benin, Benin, found that 89% of all pregnant patients with hemodynamic instability and 88% of cases of tubal rupture have ruptured EP [4].

The incidence of EP has tripled in most industrialized countries over the past 3 decades, and the annual incidence rates vary between 100 and 175 per 100,000 women age between 15 and 44 [5]. The incidence rates of EP in African countries range between 0.5 and 2.3% of live births [6]. In Ethiopia, the magnitude of EP among total deliveries was 0.82% [7]. Globally, EP accounts for almost 2% of all pregnancies, 0.25-2% of all pregnancy-related complications, and 9% of all pregnancy-related deaths [8, 9].

EP is diagnosed with a “classic” triad of delayed menstruation, pain, vaginal bleeding or spotting, and a positive pregnancy test. However, only 50% of patients present with typical symptomatology [10]. The exact etiology of EP is elusive but the factors which predispose women to EP include age, abortion status, ovulation stimulation, pelvic infection, tubal surgeries, and use of modern contraceptives [8, 11]. For example, a ten-year cross-sectional study in Nigeria (2002-2011) showed that the majority of cases were between 26 and 30 years, were married, multiparous, and had a previous history of induced abortions [11].

The clinical presentation of EP varies from asymptomatic to acute abdominal pain and may present with hypovolemia and shock [8, 11]. A four-year cross-sectional study conducted in South Africa showed abdominal pain was the main complaint of patients, accounting for 81.1% of the recorded symptoms, and more than one-third of patients had signs of shock when they presented to the hospital [12]. Most morbidity outcomes of EP included anemia, blood transfusion (78%), and wound infection (2.1%) [13]. In Ethiopia, EP accounts for 3.74% of gynecological surgeries, and the majority (51.9%) of mothers were in the age group of 25-29 years [7]. Abdominal pain, vaginal bleeding, and amenorrhea were the most common symptoms at presentation, and most mothers (61%) presented with ruptured EP [7].

EP cause has a direct impact on women's reproductive health and is a significant issue for the community [10]. EP remains a significant cause of maternal morbidity and mortality in developing countries [11]. However, the importance of EP in developing countries has received reduced attention, partly because of its late presentations of patients with ruptured EP, poor diagnostic tools, limited capacity to handle emergencies, and consequent burden of increased maternal morbidity and mortality [7]. Importantly, there is little evidence on EP in Africa in general and north Ethiopia in particular. The present study was conducted to perform a comprehensive assessment including determining the prevalence of EP, exploring management outcomes, and identifying underlying risk factors using a case of Aksum University Comprehensive and Specialized Hospital in Tigray, Ethiopia.

## 2. Materials and Methods

### 2.1. Study Design, Population, and Setting

We have performed a health facility-based retrospective cross-sectional study dated from September 2015 to August 2019. Records of total deliveries and gynecological surgeries of all pregnant mothers who were diagnosed after a clinical examination, investigations, and treatment were included in the study.

The study was conducted in Aksum University Comprehensive and Specialized Hospital (AKUCSH) and Saint Marries General Hospital in Tigray regional state, northern Ethiopia. Aksum is the capital of the central zone of Tigray which is located 1024 km north of Addis Ababa and 247 km from Mekele, the capital city of the Tigray. According to the plan and finance office of Aksum town (personal communication, 2020), the total population size is 77,723 of which 38,402 are males and 39,321 are females. Axum city has five kebeles, one referral and teaching hospital, one general hospital, two health centers, four health posts, and 10 different level private clinics.

AKUCSH started as a referral hospital in 2014 for serving an estimated 3.6 million population in its catchment areas. Currently, the hospital has around 760 health workers including 19 specialists. The hospital provides emergency, inpatient, and outpatient services at different departments. Currently, the Gynecology & Obstetrics (GYN/OBS) department has two gynecologists/obstetricians, 12 GPs, and 49 midwives. The hospital has 177 beds in four wards: medical, pediatrics, surgical, gynecology, and obstetrics wards. Of these, the maternity ward has 42 beds, and delivery and labor wards have 13 beds. Saint Marries General Hospital, the other study setting, was established in 1961 and has 368 health care workers including 17 general practitioners (GPs). In the GYN/OBS department, there are one gynecologist/obstetrician, one Integrated Emergency Surgeon and Obstetrician (IESO), and 18 midwives. The GYN/OBS department has 28 beds and delivers similar services to AUCSH.

All total deliveries and gynecological surgeries to Aksum University Comprehensive Referral Hospital and Saint Marries General Hospital from September 1, 2015 to August 6, 2019 were reviewed.

### 2.2. Variables and Data Collection Process

In the dependent variable of the study presence of favorable/unfavorable outcomes of EP, Elements of management outcomes included recorded outcomes including side effects of EP, hemoperitoneum collection, estimation of blood loss, blood transfusion, postoperative complications, duration of hospital stay, and maternal outcomes. An unfavorable outcome was assumed if one of the following identified: unstable vital sign, blood transfusion, prolonged hospital stay, postoperative complications, and death. Anemia was assumed hemoglobin value of <10.5 mg/dl. Unstable vital sign was assumed if one of the following occurred: SBP < 90 mm Hg and/or DBP < 60 mm Hg, pulse rate > 100 beats per minute, respiratory rate > 30 breaths per minute, and/or temperature > 37.5°C. A kebele is the smallest administrative unit of Ethiopia, similar to a ward, a neighborhood or a localized, and delimited group of people. It is part of districts, itself usually part of a zone, which in turn are grouped into one of the ten regions or two chartered cities that comprise the Federal Democratic Republic of Ethiopia [14]. Each kebele consists of at least 500 families or the equivalent of 3,500 to 4,000 persons. There is at least one in every town with more than 2,000 populations. A district representative had jurisdiction over to ward [15].

EPs were managed in the hospitals by laparotomy (most cases with pfannenstiel incision, minilaparotomy never tried, and medical management not available) and if patient had pneumonia complications, spinal anesthesia (SA), and prophylactic antibiotics that are routinely given. Data were retrieved on EP status, parity, clinical presentation, risk factors, findings at laparotomy, and the outcome of the treatment from the maternity ward, labor, and delivery ward and operation room registration books and medical records. Records that did not include the EP status and one of the aforementioned management outcomes were excluded from data analysis. In addition, we extracted data on relevant sociodemographic characteristics, parity, abortion, sexually transmitted infection, contraceptive use, obstetric and gynecologic surgery, and previous EPs.

The data extraction sheet was prepared in English and then translated into the local language (Tigrigna) and back to English by a professional translator. To establish face validity and translation quality, the data extraction sheet was tested on 5% of the total sample size determined for this study (participants were outside of study site) by data collectors and supervisors during training. A few questions, language clarity, and information were revised, and the data extraction sheet was finalized for the study.

Data were extracted by four BSc nurses and supervised by one GP. We delivered training for data collectors and supervisors on the study's objective, ethical procedures, and data collection process.

### 2.3. Data Analysis Process

Data were initially checked manually for completeness and consistency by the supervisor and principal investigator during the fieldwork and rechecked before data entry. Then, data were coded, entered, and cleaned using Epi Info version 7 to export to SPSS version 20.0 software for analysis and interpretation.

Descriptive statistics were performed, and data were described using tables. We applied bivariate and multivariate logistic regression analysis to identify variables associated with the management outcomes of EP. Variables with a *p* < 0.25 in the bivariate logistic regression analyses were entered into the multivariate logistic regression analyses. Chi-square tests were applied before bivariate logistic regression to check variables with small cell sizes to merge into related categories. Goodness of fit tests was conducted using Hosmer and Lemeshow Chi-square tests. Multicollinearity was assessed using collinearity diagnosis to test correlation among predictor variables. Finally, independently associated variables were considered if *p* < 0.05 in multiple logistic regression models. The strength of association was described using the adjusted odds ratio (AOR) and 95% confidence interval (CI).

## 3. Results

### 3.1. Sociodemographic and Gynecologic Characteristics of EP Women

Table 1 describes the sociodemographic and gynecologic characteristics of the study participants. The mean age of the women diagnosed with EP was 29 years (SD ± 5.96) years ranging between 19 and 45 years. A significant proportion of women (42%) with EP was in the age group of 26 to 30 years, and half (50.6%) of the study participants came from the rural area. Of the total 79 women admitted with EP, about 39% of them were multipara, 8% had a previous history of EP, and 4% had previous Caesarean section history.

### 3.2. Clinical Presentation of Women Diagnosed with Ectopic Pregnancy

Table 2 presents the clinical presentation of women diagnosed with EP. Three quarters (76%) of the women diagnosed with EP presented after 24 hours of the onset of their complications. Two thirds (65%) of them presented with stable vital signs at presentation, almost all with abdominal pain (98%) and approximately 1 in 10 with vomiting (13%). Anemia was also diagnosed in more than half (58.2%) of the women with EP.

### 3.3. Prevalence of Ectopic Pregnancy Out of Total Deliveries

The trends of EP over time are presented in Table 3. A total of 15,204 deliveries and 1043 gynecological surgeries were recorded in both hospitals from 2015 to 19. Eighty-three patients were admitted with the diagnosis of EPs but records of 79 of the patients were eligible for data analysis. The overall prevalence of EP was found to be 0.52% (79/15,204) deliveries, 1 : 193 deliveries. The surgery for EP accounts for 7.6% (79 of 1,043) of all gynecological surgeries. The highest prevalence of 0.72% (20 out of 2779) was documented in 2015, and the lowest was 0.42 (16 out of 2450) recorded in 2017.

### 3.4. Management Outcomes of Ectopic Pregnancy

Table 4 presents details of the management outcomes of EP. Among the different EP implantation sites, most cases (92.4%) occurred in the fallopian tube, followed by 5.1% in the ovary, and 2.5% in abdominal EPs. Surgical management was undertaken for all the 79 women diagnosed with EPs, including laparotomy (68.4%), salpingo-oophorectomy (17.7%), salpingectomy (8.9%), oophorectomy (2.5%), and cornual resection (2.5%). The intraoperative finding was intact tube in 47 (59.5%) cases and ruptured in the rest but the condition of EP procedure was ruptured for about two-thirds (63.3%) of the women.

Most patients (67.1%) were admitted for less than a week. Thirty (38%) patients had developed some complications after surgery including anemia (*n* = 12), fever (*n* = 10), wound infection (*n* = 2), and pneumonia (*n* = 2).

### 3.5. Factors Associated with Management Outcome of Ectopic Pregnancy

Residence (urban or rural), previous history of abortions, previous history of surgery, presentation of symptoms from the onset of illness, vital signs at presentation, gestational age, and level of hemoglobin at presentation were the candidate variables for multiple logistic regressions. Only residence and level of hemoglobin at presentation were found to be statistically significant EP management outcomes. Mothers who came from urban areas were 11 times (AOR = 11.2, 95% CI: 2.7-47.2) more likely to have favorable outcomes than those coming from rural areas. Mothers who presented with normal hemoglobin were about 10 times (AOR = 9.9, 95% CI: 2.03-48.7) more likely to have a favorable outcome than mothers presented with anemia (see Table 5).

## 4. Discussion

The present study determined the prevalence, key management outcomes, and factors associated with the management outcomes of EPs treated in Central Tigray region of Ethiopia. We found that the prevalence of EP was recorded in 5 : 1000 deliveries (0.5%), a finding consistent with studies in Guinea (0.41%) [9], and Papua New Guinea (0.6%) [16]; although different globally, EP accounts for almost 2% [8, 9], South Africa (2.2%) [12], and Nigeria (1.1%) [13]. These variations could be linked to differences in environmental conditions (e.g., exposure to toxic materials), the prevalence of related infections (e.g., sexually transmitted infections), and social lifestyle (e.g., smoking). Evidence shows that smoking and infection could affect the transportation of an oocyte and embryo through the tube by decreasing the density of cilia.

The majority of women with EP (41.8%) were in the 26-30 years age group. This is comparable with the findings of studies conducted in India (71%) and Nigeria (36%) [3, 17]. This could partly be because of the maximum level of sexual activity and fertility of the women in this age period. This connotes the special need to have regular sexual and reproductive health check-ups in these age groups. The present study found that pelvic infection was observed in 24 women diagnosed with EP and had the following common symptoms: abdominal pain, vaginal bleeding, and Amenorrhea. This is similar to the study conducted in Ethiopia [7, 18, 19]. Prevention, or at least diagnosis and treatment, of pelvic infections would warrant primary care visits. Few other identified risk factors are modifiable.

More than one-third of the women admitted with EP (38%) had an unfavorable outcome in the present study, and this was higher than a study conducted in Adigrat, Ethiopia [7]. This could partly be due to the differences in the ways that health facilities are set up in the two localities: Adigrat hospital was established many years before both hospitals in the current study setting and is also nearer to Ayder/Mekele hospital, the most equipped hospital in the region.

In this study high rate of oophorectomy (21.1%), this is similar to the study conducted in Ethiopia [20] and Boston Medical Center [21]. This high rate of oophorectomy was due to inability to achieve hemostasis without removal of ovary,

Urban residence and normal hemoglobin levels at presentation were the enabling factors linked with favorable outcomes of EP management in the present study. About 90% of hospitals in Ethiopia are located in urban areas despite more than 80% of the population living in rural areas, and it is unfortunately not surprising to see urban resident women having a more favorable outcome. Furthermore, the level of literacy among women living in urban areas is far better than their rural counterparts, and this may influence the early presentation to EP care and treatment, which is supported by research in Ethiopia and India [8, 18]. Consistent with other findings [1, 22], women diagnosed with EP and anemia were also at higher risk of unfavorable EP outcome management. Evidence suggest that endometriosis [23] and PID [24], leading to anemia, are known risk factors for adhesions and ectopic pregnancy. Additionally, the terminal ileum, sigmoid colon, and caecum were the parts of the gastrointestinal tract most commonly involved. The rectum was the source of bleeding in only one case.

The current study set out to comprehensively assess the magnitude of EP, its management outcomes, and related factors for EP management outcomes. However, there were some limitations that need highlighting. First, there was some data incompleteness linked to incomplete information, loss of patient cards, and illegible handwriting on patients' cards, gynecology registration books, and operation room records. Concerning this, we could not assess the details of sociodemographic variables and some important variables for some patients. Second, given it was a health facility-based study, findings of the study may not be generalizable to the general population. Third, because the sample size was small (*n* = 79), some of the confidence intervals in the bivariate and multivariate logistic regression were rather wide. Fourth, the fact that women do not seek medical care in Ethiopia for many pregnancies in the first trimester limits the measurement, and that clinical or chemical pregnancy rate is not generally available, while surgeries for ectopic pregnancy and deliveries are much easier to identify.

## 5. Conclusions

The magnitude of EP in our study was similar to the global rate but more than one-third of women with ectopic pregnancies had an unfavorable maternal outcome. The delayed presentation with ruptures causes a major challenge to the reproductive performance of women and leads to maternal morbidity and mortality. This calls for an early checkup to improve the diagnosis, care, and treatment of EP and its management outcomes in developing countries like Ethiopia. Women living in rural areas and having anemia during pregnancy should seek special attention in the management of EP. We also recommend improving the data management of hospitals. The health care providers should document patients' information clearly on their cards. A large scale study with primary data of adequate sample size is required to assess the full picture of EP.

## Figures and Tables

**Table 1:** Sociodemographic and gynecologic characteristic distribution of women admitted with EP in Aksum (AKU CHS CSH and Saint Marries General Hospital) in Tigray, North Ethiopia.

Variables		Frequency, *n* = 79	Percent (%)
Age	≤20 years	9	11.4%
	21–25 years	11	13.9%
	26 – 30 years	33	41.8%
	31–35 years	14	17.7%
	≥36 years	12	15.2%
Residence	Urban	39	49.4%
	Rural	40	50.6%
Ethnicity	Tigrai	77	97.5%
	Amhara	2	2.5%
Religion	Orthodox	77	97.5%
	Muslim	2	2.5%
Parity	Nulliparous	22	27.8%
	Primipara	26	32.9%
	Multipara	31	39.2%
Previous history of abortion	No abortion	47	59.5%
	History of one or two abortions	28	35.4%
	Three and above abortion	4	5.1%
Previous history of EP	No history of EP	73	92.4%
	One previous EP	6	7.6%
Previous history of pelvic infections	No history of pelvic infection	55	69.6%
	Had a history of pelvic infection at least once	24	30.4%
Previous history of surgery	No history of surgery	67	84.8%s
	Had a history of surgery at least once	12	15.2%
History of contraceptive use	No history of contraceptive use	38	48.1%
	History of contraceptive use at least once	41	51.9%

**Table 2:** Clinical presentation of patients of women admitted with ectopic pregnancy in Aksum (AKU CHS CSH and Saint Marries General Hospital) in Tigray, North Ethiopia, (*n* = 79).

	Characteristics	Frequency	Percent (%)
Time of presentation	Within 24 hours of the onset of illness	19	24.1%
	More than 24 hours of the onset of illness	60	75.9%
Vital sign at presentation	Stable	51	64.6%
	Unstable	28	35.4%
Presenting symptoms^∗^	Abdominal pain	77	97.5%
	Vaginal bleeding	64	81%
	Amenorrhea	45	57%
	Vomiting	10	12.7%
Physical examination finding^∗^	Pale conjunctiva and/or palm	34	43%
	Abdominal tenderness	75	94.9%
	Cervical motion tenderness	24	30.4%
	Adnexal mass	2	2.5%
	Adnexal tenderness	12	15.2%
Urine HCG result	Positive	77	97.%
	Negative	2	2.5%
Level of hemoglobin at presentation	Hemoglobin > 10.5 at presentation	33	41.8%
	Hemoglobin < 10.5 at presentation	46	58.2%
Diagnosis	Clinical only	4	5.1%
	Ultrasound only	2	2.5%
	Intraoperative only	2	2.5%
	Clinical and ultrasound	71	89.9%
Gestational age	Less than 7 weeks	45	57%
	7–9 weeks	18	22.8%
	More than 9 weeks	16	20.2%

^∗^Participants reported more than one symptom or sign.

**Table 3:** Yearly distribution of ectopic pregnancy per number of registered deliveries in Aksum (AKUCSH and Saint Marries General Hospital), Tigray, Ethiopia, 2015-19.

Period	Total registered deliveries	Total EP	Percent (%)
2015	2779	20	0.72
2016	3169	17	0.54
2017	3761	16	0.42
2018	5495	26	0.47
Total	15204	79	0.52

EP: ectopic pregnancy.

**Table 4:** Management outcome patients admitted with ectopic pregnancy in Aksum, Tigray, North Ethiopia (*n* = 79).

	Characteristics	Frequency	Percent (%)
Side of EP	Right side	47	59.5%
	Left side	32	40.5%
Condition of EP	Ruptured	50	63.3%
	Unruptured	29	36.7%
Hem peritoneum collection	Less than 500 ml	34	43%
	≥500 ml	18	22.8%
	No hem peritoneum	16	20.3%
	Not documented	11	13.9%
Estimated blood loss	Less than 500 ml	28	35.4%
	500 ml–1000 ml	10	12.7%
	More than 1000 ml	6	7.6%
	Not documented	35	44.3%
Transfuse for blood	Not transfused	52	65.9%
	2 units	20	25.3%
	3 units	5	6.3%
	4 units and above	2	2.5%
Postoperative complications	No complication	49	62%
	Anemia	12	15.2%
	Fever	10	12.7%
	Wound infection	6	7.6%
	Pneumonia	2	2.5%
Duration of hospital stay	Less than 7 days	53	67.1%
	7-14 days	24	30.4%
	14 days–1 month	2	2.5%
Maternal outcome	Favorable	49	62%
	Unfavorable	30	38%

**Table 5:** Factors associated with maternal outcome among women admitted with ectopic pregnancy in Aksum, Tigray, North Ethiopia (*n* = 79).

Variable	EP management outcome		COR 95% CI	AOR 95% CI
	Favorable	Unfavorable		
Residence				
Rural	17	23	1	1
Urban	32	7	6.19 (2.21-17.3)	11.2 (2.7-47.2)^∗∗^
History of abortion				
No	26	21	1	1
Yes	23	9	2.06 (1.07-5.4)	4.25 (1.03-19.5)
Vital sign				
Unstable	12	16	1	1
Stable	37	14	3.52 (1.34-9.3)	1.56 (0.4-5.5)
History of surgery				
Yes	6	6	0.56 (0.2-0.9)	2.03 (0.4-10.9)
No	43	24	1	1
Hemoglobin				
Anemic	22	24	1	1
Nonanemic	27	6	4.9 (1.7-4.1)	9.9(2.03-48.7)^∗∗^
Presentation of illness				
Within 24 hr	10	9	0.6 (0.2-0.9)	0.3 (0.06-1.3)
More than 24 hr	39	21	1	1
Gestational age				
< 7 weeks	31	14	2.2(1.6-7.1)	1.2 (0.2-6.7)
7-9 weeks	10	8	1.3 (1.3-4.8)	1.7 (0.2-13.5)
> 9 weeks	8	8	1	1

^∗^For variables showing significant association during multivariate analysis at *p* < 0.05.

## Data Availability

The datasets analyzed in the study are available from the corresponding author upon request.

## Abbreviations

ANC: Antenatal care
BP: Diastolic blood pressure
C/S: Cesarean section
DES: Diethylstilbestrol
E.C: Ethiopian calendar
EDHS: Ethiopian Demographic Health Survey
EP: Ectopic pregnancy
GP: General practitioner
GYN/OBS: Gynecology and obstetrics
HCG: Human chorionic gonadotrophic hormone
HIV: Human immunodeficiency virus
HP: Heterotopic pregnancy
IUD: Intrauterine device
OC: Oral contraceptives
°C: Degree Celsius
Km: Kilometer
M: Meter
PID: Pelvic inflammatory disease
PNG: Papua New Guinea
RH: Reproductive health
RVI: Retroviral infection
SD: Standard deviation
STDs: Sexually transmitted diseases
SPSS: Statically Package for Social Science
TVUS: Transvaginal ultrasound
UK: United Kingdom
U/S: Ultrasound
WHO: World Health Organization.

## Supplementary Materials

Supplementary Materials Questionnaire 1.
