# Supplementary material for: Ectopic Pregnancy in Tigray, Ethiopia: A Cross-Sectional Survey of Prevalence, Management Outcomes, and Associated Factors
Source: J Pregnancy. 2021 Nov 30;2021:4443117. doi: 10.1155/2021/4443117 (PMC8651379; doi:10.1155/2021/4443117)
Supplement: Supplementary Materials — Questionnaire 1. [file 4443117.f1.docx]

**QUESTIONNAIRE**

**Questionnaire format:** To be filled for those who had ectopic pregnancy in Aksum university comprehensive and specialized hospital and St marry general hospital.

I am serving as a data collector for a the study done on prevalence and management outcome of ectopic pregnancy conducted in AKUCSH and St. marry general hospital by fifth year medical student of Aksum university. This is to collect data from patient records in AKUCSH; a four-year facility based cross-sectional retrospective study.

Data collector name and signature ______________________________________

**Instruction:** Please encircle the letter corresponding to the correct record or write the correct record on the space provided.

- 1. **IDENTIFICATION**

1. Questionnaire I.D: _______________
2. Card No_______________________
3. Date of admission­­­­________________
4. Date of discharge________________
5. **SOCIO DEMOGRAPHIC CHARACTERISTICS**
6. Age in year ____________________
7. Marital status
8. Single B. Married C. Divorced D. Widowed E. not documented
9. Residence
10. Urban B. Rural C. not documented
11. Educational status (Grade completed)
12. Unable to read and Write B. 1-6^th^  C. 7-12^th^ grade E. College or University F. not documented
13. Ethnicity
14. Tigraweyti B. Oromo C. Amhara D. Other______ E. not documented
15. Religion A. Orthodox B. Muslim C. Catholic D. Other _______ E. Unknown
16. **PREVIOUS GYNECOLOGICAL HISTORY**
17. Pervious history of abortion
18. 1 B. 2 C. 3 and above D. No history
19. Had the women had previous ectopic pregnancy?
20. 1 B. 2 C. 3 and above D. No history
21. Had the women had previous surgery?
22. Tubal ligation B. Cesarean section C. Appendectomy

D. Tubal anastomosis E. previous ectopic F. Other__________ G. No history

1. Had the women had previous genital tract infection?
2. Genital ulcer B. Vaginal discharge C. PID D. Known RVI E. No history

F. Other__________

1. Had the women had previous contraceptive use?
2. IUCD B. Depo-Provera C. OC pills D. Barrier E. No contraceptive used

F. Other__________

1. Had the women had previous treatment for infertility?
2. ART B. Ovulation induction C. Other ________D. no history
3. **PREVIOUS OBSTETRICS HISTORY**
4. Gravidity
5. Primi gravida B. 2-4 C. > 5 D. Unknown E. not mentioned
6. Parity
7. 0 B. 1 C. 2 D. 3 E. 4 F. > 5. G. not mentioned
8. Had the women had previous caesarean section
9. 1 B. 2 C. 3 and above D. No history E. not mentioned
10. **PRESENTING FEATURES OF ECTOPIC PREGNANCY**
    - 1. Presentation of symptoms
      2. within 24 hour B. more than 24 hour C. not mentioned
      3. Clinical features of ectopic pregnancy from history
      4. Abdominal pain B. Vaginal bleeding C. Amenorrhea D. vomiting

E. dizziness or fainting F. Other _________

- - 1. Vital sign at presentation
    2. Stable B. Un stable
    3. Physical finding of the patient at presentation
    4. Pallor B. Abdominal tenderness C. Cervical motion tenderness D. Adnexal mass E. Adnexal tenderness F. Other _________
    5. Result of Urine HCG
    6. Positive B. Negative C. Unknown
    7. Hemoglobin level at presentation
    8. > 10.5 mg/dl B. 5-10.5 mg/dl C. < 5mg/dl D. Unknown
    9. Diagnosis of ectopic pregnancy
    10. Clinically B. U/S C. Culdocentesis D. Intraoperative
    11. Gestational age (with LNMP, physical examination, or U/S)
    12. < 7 Weeks B. 7- 9 Weeks C. >9 Weeks D. not documented

1. **MANAGEMENT OF ECTOPIC PREGNANCY**
2. Medical
3. Yes if yes, specify the type of therapy_______ B. No
4. Surgical
5. Salpingectomy B. Salpingo-oophorectomy C. Oophorectomy D. Courunal resection E. Hysterectomy F. Tubal ligation G. Milking H. others___________
6. Condition of ectopic pregnancy
   - 1. Ruptured B. Un-ruptured C. Tubal abortion D. Chronic ectopic E. Heterotopic pregnancy F. Other _________
7. Hem peritoneum collection
8. < 500 ml B. > 500 ml C. no hem peritoneum collection D. not mentioned
9. Estimated of blood loses record A. Yes if yes, specify in ml_________ B. No record
10. Site of ectopic pregnancy
11. Fallopian tube.
12. Ampulla 2. Isthmic 3. Courunal 4. Fimbria 5. not mentioned
13. Ovarian
14. Cervical
15. Unknown
16. Side of ectopic pregnancy A. Right B. Left C. Unknown
17. Did the women transfused for blood?
18. 1 Unit B. 2 Unit C. 3 Unit D. > 4 Unit E. No transfusion
19. Hospital stay
20. < 7 days B. 7-14 days C. 14 days -1month D. > 1 month
21. Postoperative complication
22. Fever B. Wound infection C. Pneumonia D. Anemia E. Other______ F. No complication
23. Did the women die?
24. Yes if yes, specify the cause of death ___________
25. No
